# Experimental Study and Mathematical Modeling of a Nanofiltration Membrane System for the Recovery of Polyphenols from Wine Lees

**DOI:** 10.3390/membranes12020240

**Published:** 2022-02-18

**Authors:** Alexis López-Borrell, María-Fernanda López-Pérez, Salvador Cayetano Cardona, Jaime Lora-García

**Affiliations:** Instituto de Seguridad Industrial, Radiofísica y Medioambiental (ISIRYM), Universitat Politècnica de València (UPV), Plaza Ferrándiz y Carbonell, s/n, 03801 Alcoy, Spain; allobor1@epsa.upv.es (A.L.-B.); scardona@iqn.upv.es (S.C.C.); jlora@iqn.upv.es (J.L.-G.)

**Keywords:** nanofiltration, polyphenols, wine lees, polyphenols recovery, Spiegler–Kedem model

## Abstract

The winemaking process in Spain generates a significant amount of wastes such as wine lees. Currently, the nanofiltration process is a viable technique for the revalorization of compounds from wastes. In this aspect, this technique can be used for the recovery of compounds, such as polyphenols, as well as active principles widely used in industries, such as pharmaceuticals or cosmetics. Polyphenols are found in acceptable amounts in wine lees wastes and it is interesting to study the nanofiltration process viability to recover them. In order to study this possibility, it is necessary to determine the choice of the best membrane to use and the effect of operational parameters such as pressure, temperature, cross-flow rates, and concentration. In addition, it is important to be able to develop a mathematical model that can help in the future design of lees treatment plants. The treatment of red wine lees to concentrate polyphenols has been studied in a laboratory plant using different membranes (RO and NF) at different pressures (4.5, 9.5, and 14.5 bar), different temperatures (293, 303, and 308 K), and two concentrations (2100 and 1100 mg tyrosol eq·L^−1^). The results have been encouraging to consider nanofiltration as a viable technique for the treatment and revalorization of this waste. The most suitable membrane has been the NF270, in which 96% rejection rates have been obtained, with a flux of 30 L·h^−1^·m^−2^. Moreover, in this study, the Spiegler–Kedem model (SKM) was used to calculate mass transfer constants and permeabilities. Suitable adjustments of these parameters were obtained to validate this mathematical model. For this reason, the SKM might be used in future studies to continue in the research work of the treatment of wine lees wastes.

## 1. Introduction

Currently, wine producers and wine consumers are increasingly aware of the environmental impact of this industry. Therefore, this sector is extensively searching for new processes to reuse solid wastes or wastewater in order to adapt to climate change, minimize environmental loads, and become a modern wine production process, especially in dry areas such as Spain [1].

In this way, there is an interest in the recovery of the wine residues generated during its production. Among these products are natural antioxidants, there are a lot of phenolic compounds present in winery wastes, and, in addition, they are considered completely safe compared to synthetic antioxidants [2]. These polyphenols have beneficial effects, including the scavenging of free radicals, antioxidants, and chelation of transition metals and anti-inflammatory properties.

Due to the properties of polyphenols and their concentration in wine wastes, pharmaceutical, food, and cosmetic industries are considering their recovery and purification to obtain new products with high added value [3,4,5,6,7].

These important compounds are present in all grape parts and some of them are extracted in wine. Nevertheless, many of these products remain with a considerable concentration in vinification wastes such as pomaces, stems, seeds, and fermentation wastes (wine lees).

Wine lees are defined as “the residue that forms at the bottom of vessels containing wine, after fermentation, during the storage or after authorized treatments, as well as the residue obtained following the filtration or centrifugation of this product” (European Economic Community (EEC) regulation No. 337/79). The polyphenol concentration present in this residue is enough to consider its revalorization [8]. Currently, wine lees are the main source of commercial tartaric acid production, which is widely applied in the food, pharmaceutical, and chemical industry. Tartaric acid production includes precipitation, acidification, extraction, crystallization, distillation, ion exchange, and adsorption processes. However, polyphenols are destroyed in these chemical and heating treatment steps thus membrane filtration can be a feasible technology to obtain these active principles. 

Nowadays, due to their high selectivity, membrane processes can be considered a suitable technology to separate bioactive compounds such as proteins or vitamins [9]. In the case of the wine industry, one of the uses of membrane filtration is wine dealcoholization [10]. Moreover, there is a growing interest in the use of membranes to recover and purify phenolic compounds from the wine lees. Other advantages of this membrane technology are its easy scale-up and modularity, the low requirements of energy, and its environmental safety [11]. Many research papers have been published in the last few years containing the keywords polyphenol, recovery, and wine. In this sense, 291 publications can be retrieved from the Web of Science Core Collection using those keywords, focusing on journals related to food science and technology. However, when membrane is added as a keyword, only 21 publications are recovered. Additionally, when the keyword lees is included in this literature search, only eight references are found, as shown in Table 1.

When this methodology is proposed as a separation system, preliminary studies, regarding the choice of membrane type and material, operational conditions, and interactions of the solutes from the feed solution with the membrane, must be done [12]. In this context, combinations of different types of membranes can be interesting to achieve higher performance. Microfiltration (MF) and Ultrafiltration (UF) processes could be used to eliminate organic compounds with high molecular weight and less commercial use, and, later, phenolic compounds from wine lees could be concentrated with Nanofiltration (NF) and Reverse-osmosis (RO) techniques. In recent years, NF and RO have gained widespread interest and have become widely adopted as modern tools for this type of industrial application [13,14,15,16,17]. Therefore, an optimal design is necessary to implement a membrane system in an industrial process. The development of a mathematical model is mandatory to achieve an optimal design. This model must adequately describe the performance of the membrane process for improving its efficiency and lower its cost. On the other hand, such a mathematical model should be as simple as possible and with the fewest number of parameters, requiring the least possible number of experiments to save money and time in its implementation. Concerning nanofiltration mathematical models, some take into account the transport mechanism, such as the Nernst–Planck model [18]. Another model, such as the Spiegler–Kedem model (SKM), predicts the transport of solute and solvent regardless of the type of solute and its charge, solvent, and membrane [19,20].

The SKM mathematical model states that solute ($J_{s}$ and L·m^−2^·h^−1^) and solvent fluxes ($J_{v}$ and L·m^−2^·h^−1^) are due to chemical potential gradients across the membrane. This chemical potential is caused by a concentration (solute transport) or pressure (solvent transport) gradient [21]. Membranes in this model are considered as a black box, which is characterized by solute permeability ($P_{s}$ and L·m^−2^·h^−1^), solute reflection coefficient (σ), and mass transfer coefficient (*k* and L·m^−2^·h^−1^) parameters. The SKM is based on irreversible thermodynamics to describe the transport when the membrane structure and transport mechanism are not fully understood. This model usually is applied when electrostatic interactions between the solute and the membrane are negligible because the membrane is uncharged or the solute is neutral. In this work, it is considered that the osmotic pressure gradient (Δπ, bar) is mainly caused by polyphenols’ concentration and thus the simplified equations of the SKM model are as follows:

(1) $$J_{v}=L_{p}\cdot \left(\Delta P-\sigma \Delta \pi \right)$$

(2) $$J_{s}=P_{s}\cdot \left(C_{m}-C_{p}\right)+\left(1-\sigma \right)\cdot C_{m}\cdot J_{v}$$

where $C_{m}$ and $C_{p}$ are polyphenol concentrations at the membrane surface and permeated flow, respectively, expressed in mol·L^−1^, and $L_{p}$ is the hydraulic permeability of the membrane (L·m^−2^·h^−1^·bar^−1^). Due to $L_{p}$ as a parameter dependent on temperature, an Arrhenius equation is used to describe this behavior. ΔP is transmembrane pressure in *TMP* (bar).

In developing the model the same way as other authors [22], the following expression of the permeate flux ($J_{v}$, L·m^−2^·h^−1^) is found with the parameters above indicated:

(3) $$J_{v}=L_{p0}\cdot exp\left(-\frac{\Delta H}{R}\cdot \left(\frac{1}{T}-\frac{1}{T_{0}}\right)\right)\cdot \left[\Delta P-\sigma \cdot R\cdot T\cdot \left(C_{f}-C_{p}\right)\cdot exp\left(\frac{J_{v}}{k}\right)\right]$$

where $L_{p0}$ (L·m^−2^·h^−1^·bar^−1^) is the pre-exponential coefficient, Δ*H* is the dissolution enthalpy in the membrane (J·mol^−1^), R is the universal gas constant (J·mol^−1^·K^−1^), and $C_{f}$ (mol·L^−1^) is the polyphenols’ concentration in the feed solute. *T*_0_ and *T* (K) are reference and process temperatures, respectively.

The main object of this work is to research the feasibility of nanofiltration technology and the SKM model for the treatment of wine lees to recover polyphenols, studying several operational conditions such as pressure, feed concentrations, and temperature. The validation of the model using experimental data results from wine lees experiments allows for obtaining important parameters useful for membrane process assessment and prediction to provide a sound basis for further design and development of this process.

For this purpose, a non-linear regression procedure has been implemented in Matlab R2020b The MathWorks, Inc. (Natick, MA, USA) based on the Levenberg–Marquardt algorithm for estimating the fitting parameters of the mathematical models applied.

## 2. Materials and Methods

### 2.1. Wine Lees Feedstock

The wine lees effluent used in this work came from the first fermentation of red wine production (Shiraz, Cabernet-Sauvignon, Merlot, Gironet, and Monastrell variety) in a wine factory located in the north of the Alicante region in Spain. The raw feed solution was pre-filtered by conventional filtration (low-pressure pump at around 1 bar) with a 45 μm nominal pore size and the liquid filtrate was the feed to the membrane system. The polyphenol concentration considered as a significant characteristic of the wine lees samples was around 2036 mg tyrosol eq·L^−^^1^. The Folin–Ciocalteu method was conducted to determine the concentration of total phenolic compounds using tyrosol as the reference compound [23]. The main physical–chemical characterization of this solution is shown in Table 2. Two different concentrations of polyphenols were processed in the experiments, wherein C1 was the original solution while C2 was a diluted solution with a concentration of around 1100 mg tyrosol eq·L^−1^. The concentration of tyrosol and total polyphenols was similar to the concentrations obtained by other authors [24].

### 2.2. Membranes and Experimental Setup

This study used NF90, NF245, NF270 Dow-Filmtech™ DuPont de Nemours, Inc. © 2020 DuPont de Nemours, Inc. (Le Grand-Saconnex, Swiss), and ESPA1 Hydranautics Corporation (Oceanside, CA, USA) membranes with some of the specifications found in Table 3. 

**Figure 1:**
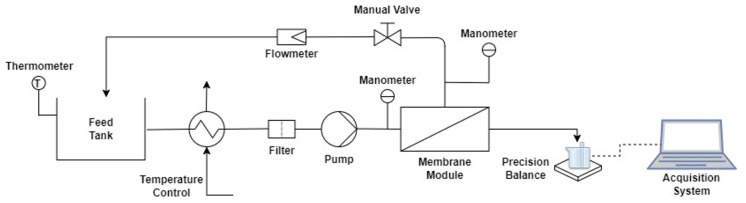
Setup of the nanofiltration plant.

The experiments were performed in a typical NF pilot plant with a flat-sheet membrane module CF042SS CELL, Sterlitech (Auburn, WA, USA) that allowed for working with one membrane with an effective area of 42 cm^2^ (Figure 1). The temperature control system was implemented by a thermostatic bath with external recirculation FRIGITERM-10, J.P. SELECTA, (Selecta, Spain).

The pilot plant had a data acquisition system for monitoring temperature as well as module input and output pressure, programmed with LabVIEW^®^. Permeate was collected during the filtration process in a glass placed on an electronic balance connected to a computer in order to continuously register the permeate weight data with a sample time of 30 s. These weight data were used to calculate permeate flux through the membranes.

**Figure 2:**
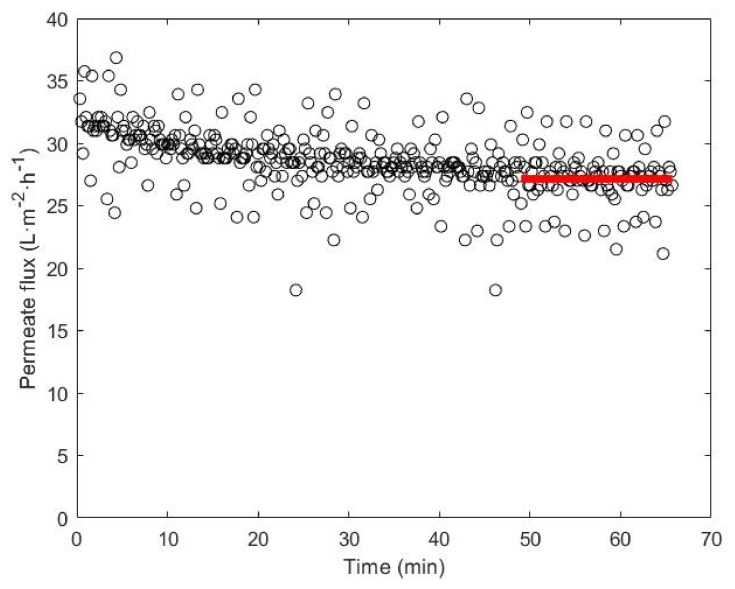
A typical experiment carried out for the tests with the membranes.

When a new membrane was selected, a hydration period of 24 h was applied for its conditioning. After membrane cleaning, its preservation was carried out with a 1% formaldehyde solution. Nanofiltration experiments were always carried out following the same procedure. In the first step, the membrane was cleaned with osmotic water, and in the second step, the membrane compaction was produced for 1 h applying the operational conditions associated with the next experiment. Then, the experiment began and, later, when the permeated flux was constant in the course of the test, an average of the points was taken as flux $J_{v}$. In Figure 2, a typical graphic is shown where the interval of time corresponding to stable experimental flux data and the average permeate flux are highlighted in red.

The membrane was conveniently cleaned after each run by flushing the pilot plant with a 10 L·min^−1^ flow rate of osmotic water kept recirculating for 30 min. This cleaning was used to remove the superficial solutes over the membrane to simulate a real industrial activity and in order to study the irreversible fouling process, chemical cleaning cycles were done (the membrane was submerged into a NaOH solution for 30 min and after water cleaning with recirculation).

Two kinds of experiments were performed in this work. On the one hand, a battery of membrane selection tests was carried out to find the best membrane in the wine lees treatment. On the other hand, the best operating conditions and the mathematical model validation were studied. Among all experiments, the criteria of the choice of the membrane was based on the permeate flux and the percentage of the retained polyphenols’ values.

The experimental conditions were set throughout the study considering the operational parameters that have the most significant impact on the performance of the experiments: transmembrane pressure (*TMP*), feed temperature, cross-flow velocity, and solution concentration. When the membrane selection was carried on four different values of *TMP*, we tested with increments of 5 bar, 4.5, 9.5, and 14.5, and, in some cases, 19.5 bar. 

Similarly, the feed temperature values were changed in 5 K increments between 293.15 K and 308.15 K. Experiments have been performed by changing the tangential velocity between a minimum and maximum velocity for each transmembrane pressure (the used velocities were 0.35, 0.42, 0.54, and 0.66 m·s^−1^). Finally, two concentrations were used with the wine lees residues: one with the original concentration (C1) and the other diluted in half (C2). 

The hydrophilicity of the membranes was measured by monitoring the wetting angle using an EasyDrop Standard goniometer, model FM140, supplied by KRÜSS GmbH (Hamburg, Deutchland), with a measurement range from 1 to 180 °C and precision of ± 0.1 °C. This equipment was supplied with a video capture kit and analysis software (Drop Shape Analysis SW21; DSA1 by KRÜSS GmbH (Hamburg, Germany). To obtain good wetting angle values that show hydrophilicity with the membrane, a sample was cut and left to dry for 24 h. The membrane sample was attached to the metal support of the equipment using adhesive tape, remaining as smooth as possible. Once the sample was placed in the equipment, a drop of osmotic water was deposited on its surface with the help of a syringe. The camera connected to the equipment focused at all times on the drop of water and the wetting angle was determined by means of the software.

## 3. Results and Discussion 

### 3.1. Membrane Selection

To choose the best membrane and check the behavior of the membranes against the phenolic compounds, it was decided to use a tyrosol solution with a concentration of 1 g·L^−1^. This compound, an essential active principle in the pharmaceutical and cosmetic industry, is present in red wine and has a low molecular weight [25].

According to the investigations of other authors [17,29,30], it has been demonstrated that the NF90 membrane is the one that offers the best rejection for low molecular weight phenolic compounds. Therefore, it was decided to perform a preliminary group of experiments with the tyrosol solution for the aforementioned experimental conditions. The rejection of the NF90 membrane to tyrosol was determined from each of the experiments carried out. The best experimental conditions were determined at a low *TMP* of 9.5 bar and minimal temperature of 293.15 K, resulting in a tyrosol rejection of 82.50% (Table 4).

**Figure 3:**
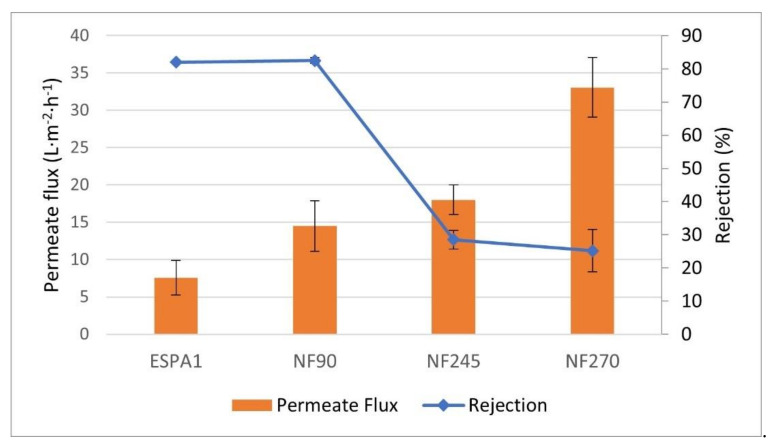
Permeate flux and rejection offered by each of the membranes to the tyrosol solution. ■ Rejection (%) and ■ permeate flux (L·m^−2^·h^−1^). *TMP* 9.5 bar, *T* 293.15 K.

Once the best operating conditions had been determined, the experiments were carried out for the other three membranes to compare their results. In Figure 3, the comparative results of the permeate flux and rejections offered by each of the membranes studied can be observed.

By observing the results of the response of the membranes to tyrosol, it can be concluded that the NF245 and NF270 membranes were the ones that provided the highest permeate flux but with very low rejection. On the other hand, the NF90 and ESPA1 membranes presented the best rejection conditions with 82.50 and 82.00%, respectively. Between these two membranes, NF90 had a higher permeate flux than ESPA1 and, therefore, the nanofiltration membrane might be the type chosen to concentrate wine lees wastes.

**Figure 4:**
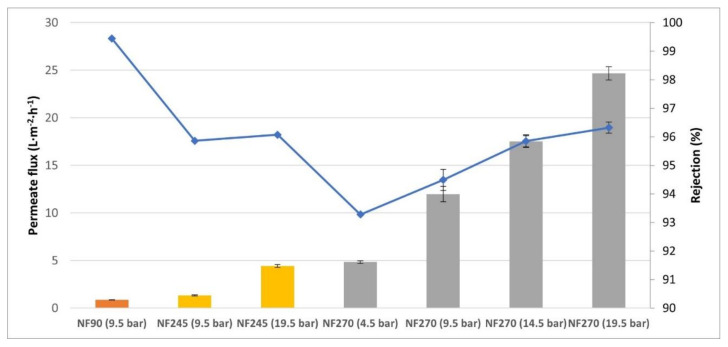
Rejection and permeate values for wine lees residues (C1) compared for nanofiltration membranes. ■ Rejection (%), ■ Permeate flux NF90 (L·m^−2^·h^−1^), ■ Permeate flux NF245 (L·m^−2^·h^−1^), ■ Permeate flux NF270 (L·m^−2^·h^−1^).

The next step of the experimental planning was the treatment of wine lees wastes with NF membranes. For a first test, the best experimental conditions determined for the solution of tyrosol were set to treat these wine lees wastes (C1 solution). It was observed (Figure 4) that for these experimental conditions, the NF90 membrane was not appropriate for these wastes since, although the rejection of polyphenols was higher than 99%, the permeate flux was too low for industrial applications (lower than 1 L·h^−1^·m^−2^). For the study carried out with the NF245 membrane, the experiment was done at identical conditions of *TMP* 9.5 bar and a temperature between 293.15 K and 298.15 K. However, the results were similar to the NF90 membrane fluxes, thus it was decided to increase *TMP* to 19.5 bar, giving an interesting permeate flux for industrial uses. Finally, the NF270 membrane was tested with different experimental conditions, offering up optimal data at *TMP* 4.5 bar and at a temperature between 293.15 K and 298.15 K. Figure 4 shows the permeate flux and rejection values obtained when different experiments were carried out under optimal operating conditions for each NF membrane.

As it can be seen in the results obtained, the NF90 and NF245 membranes presented very low permeate flux values, although the rejection of phenolic compounds was high. On the other hand, the NF270 membrane presented higher permeate flux values (around 5 L·m^−2^·h^−1^) than NF 90 and NF245, even if *TMP* was lower (4.5 bar in front of 9.5 bar, 14.5 bar, or 19.5 bar) and it a rejection of greater than 93%. The objective was to concentrate the phenolic compounds for an industrial process, thus the NF270 membrane was chosen to continue this research work, which offered acceptable permeate flux values and rejections at working pressures. Using real wine lees solutions for the industrial plant design is preferable to using a synthetic waste solution.

### 3.2. Membrane Characterization: Pure Water Permeability

The hydraulic permeability of the NF270 membrane, $L_{p}$, was determined with osmotic water in two series of experiments (series I and II). In series I, the membrane was tested before working with the problem solution, while in series II, the membrane was tested after its use with the wine lees solutions (after all experiments).

**Figure 5:**
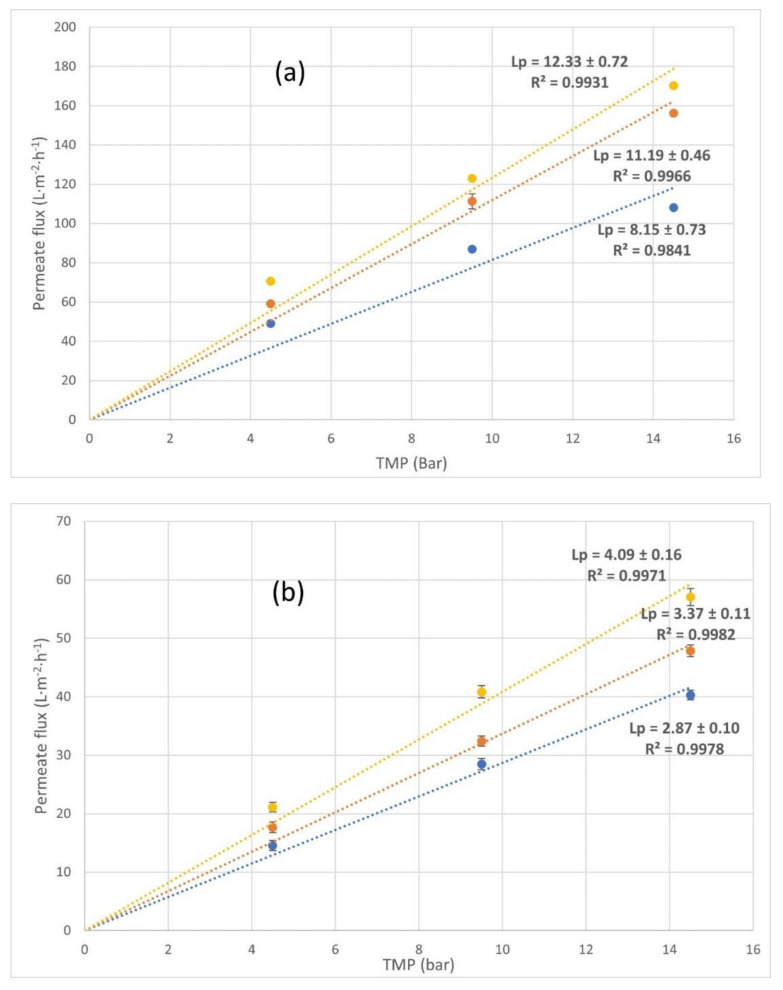
Determination of the permeability of NF270 membrane to osmotic water. (**a**) Before being exposed to lees residues (series I) and (**b**) after being exposed to lees residues and being cleaned (series II). ● permeate flux at *T* = 293.15 K (L·m^−2^·h^−1^), ● permeate flux at *T* = 298.15 K (L·m^−2^·h^−1^), and ● permeate flux at *T* = 303.15 K (L·m^−2^·h^−1^).

The results of the characterization for each of the temperatures can be observed in Figure 5. These permeability values have been obtained by adjusting Equation (1) to the experimental data (Table 5), assuming that osmotic pressure is null. Its value was deduced from the slope when the linear regression was performed, plotting the permeate flux vs. transmembrane pressure (Figure 5).

The permeability values obtained for series I (Figure 5a) were slightly higher than those indicated by the manufacturer. However, from other bibliographic references checked, it could be observed that the permeability obtained in this work was similar to the values described in the literature at a characterization temperature of 298.15 K [31,32]. These pure water fluxes have a trend with *TMP* according to Equation (1) and, in addition, there is an increase with temperature.

After each test with the wine lees wastes, the membrane was washed with osmotic water at complete recirculation using the procedure explained in Section 3.1. *Membranes* selection. Chemical cleaning was not applied in order to study how fouling affected the permeability membrane. For this reason, once the experiments were completed, the permeability of the membranes to water was measured again to observe the degree of fouling (series II). The results can be seen in Figure 5b and they show that the water permeability of the membrane was drastically reduced after wine lees residues were used. It must be considered because this fouling or superficial layer changes the water dissolution mechanism through the membrane. Figure 5 shows a suitable trend with mathematical Equation (1) when *TMP* or temperature were modified, wherein *L_p_* increases as the temperature and transmembrane pressure increase. 

As mentioned before, this reduction in the permeability of the NF270 membrane could be due to irreversible fouling by the combined action of different mechanisms. It is likely that the most relevant phenomena that occurred could be the adsorption of the organic and inorganic molecules contained in wine lees solutions, forming a superficial layer or interacting with the membrane that affected the water dissolution mechanism through the membrane. With regard to the hydrophilicity of the membrane, the wetting angle of the used membrane after water cleaning shows an increase compared to that observed in the new membranes (Table 6). The wetting angle of the droplets with the surface increases from 19.3° in a new membrane to 43.5° when this membrane is used, thus the hydrophobicity increases. In addition, a chemical cleaning was done, although there is no appreciated difference. In conclusion, the wetting angles obtained after this chemical cleaning might indicate that the fouling was irreversible.

According to the results, the degree of the irreversible fouling was determined by calculating the fouling index (FI) with osmotic water for each temperature and pressure. The results of irreversible fouling can be seen in Table 7. 

From Table 7, the degree of fouling was clearly affected by neither the temperature nor pressure changes. Nevertheless, a slight increase in the pressure decreased the degree of fouling in the membrane. This phenomenon could be due to the increase in the flow rate when *TMP* raised, which enhanced turbulence that could cause a slight cleaning of the membrane [33,34,35]. 

It is observed from the above results that the pure water permeability for the used membranes was lower than *L_p_* when new membranes were tested. The presence of the fouling compounds in the water makes the membrane surface decrease its permeability. This fact has to be taken into account in the design of the NF plants when this type of water is treated. This phenomenon of *L_p_* decreasing was indicated by Jamel Kheri-ji who concluded in his work that the membrane surface was modified due to a contraction of pores [36].

### 3.3. Influence of Experimental Parameters on the Wine Lees Nanofiltration

For the application of the industrial nanofiltration process to wine lees waste valorization, it is important to set the optimal operational conditions. In this section, several experiments were carried out to estimate the influence of these parameters over the polyphenol concentration and permeate flux.

**Figure 6:**
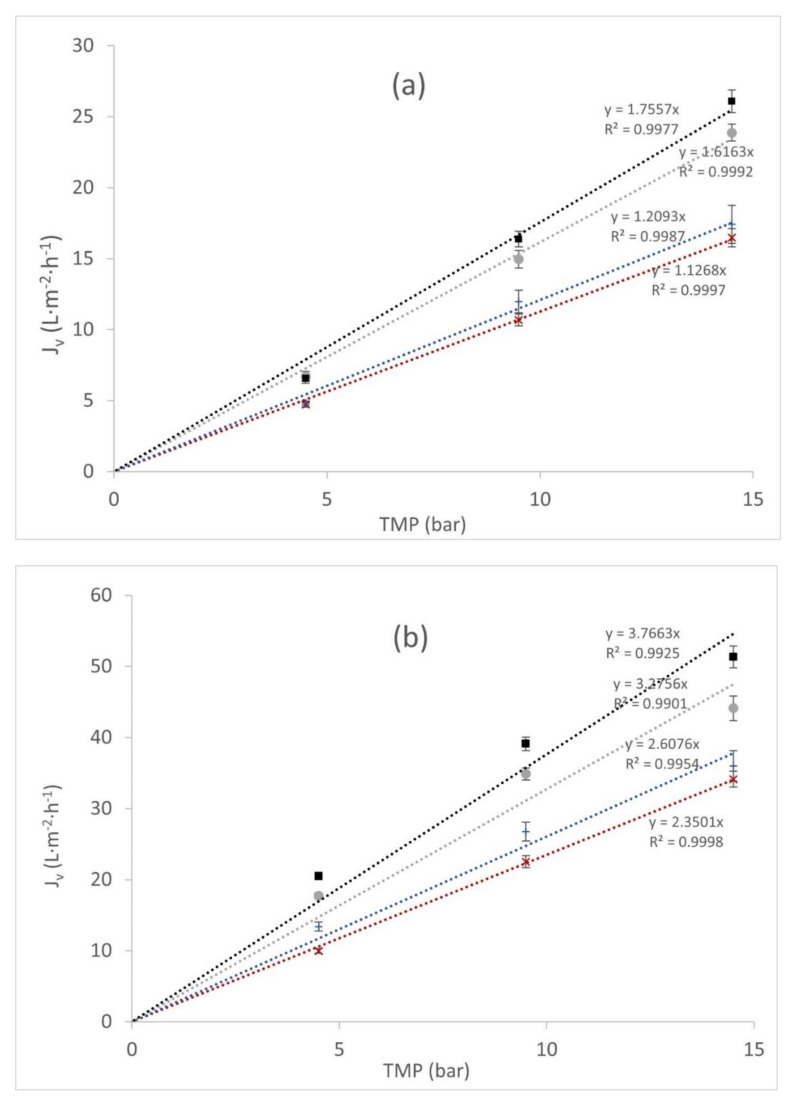
Wine lees permeate flux (*J_v_*, L·m^−2^·h^−1^) in NF270 vs. transmembrane pressure (bar) at different temperatures. (**a**) Higher lees concentration C1 (mol·L^−1^) and (**b**) lower lees concentration C2 (mol·L^−1^). ■ corresponds to *T* = 308.15 K, ● corresponds to *T* = 303.15 K, + corresponds to *T* = 298.15 K, and × corresponds to *T* = 298.15 K.

In Figure 6, the variation of permeate flux for NF270 is displayed as a function of the transmembrane pressure for NF270. Four temperatures (293.15 K, 298.15 K, 303.15 K, and 308.15 K) were studied with two different concentrations of wine lees. For both concentrations (C1 and C2 solutions), the NF270 permeation fluxes increased linearly with transmembrane pressure for all temperatures. Thus, it is supposed to maintain the concentration polarization (CP) layer due to the permeation flux not being restricted. The feed polyphenol concentration ratio (C2:C1 ≈ 1/2) was similar to the permeate flux ratio (*L_p_C1:L_p_C2*) in every operational condition.

**Figure 7:**
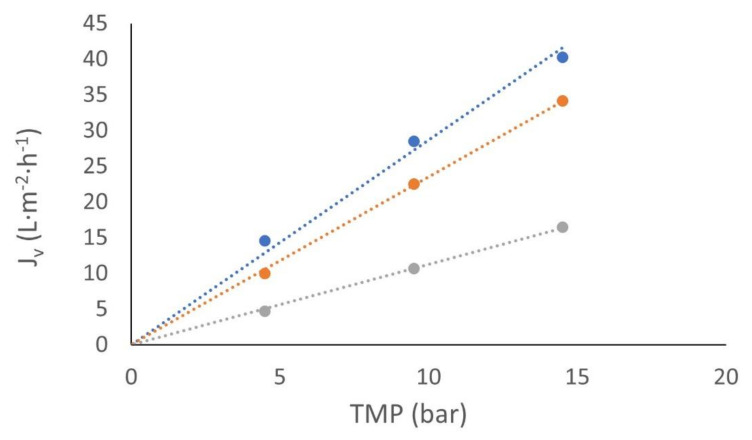
Permeate flux in NF270 vs. transmembrane pressure at 20 °C. ● *L_v_* pure water (L·m^−2^·h^−1^), ● *J_v_* with C2 polyphenol concentration (L·m^−2^·h^−1^), and ● *J_v_* with C1 polyphenol concentration (L·m^−2^·h^−1^).

However, this CP layer was sufficiently important when the polyphenol concentrations were taken into account. In Figure 7, permeate fluxes vs. *TMP* at the temperature of 293.15 K are presented. At low concentrations (solution C2), the differences between water-pure and wine lees solution permeation fluxes maintained as constant, around 20% in all temperatures. On the other hand, when high concentrations of wine lees (solution C1) were analyzed, the permeate fluxes’ differences between the pure water and solution C1 increased to 60%. In Figure 7, permeate fluxes vs. *TMP* at the temperature of 293.15 K are presented. 

**Figure 8:**
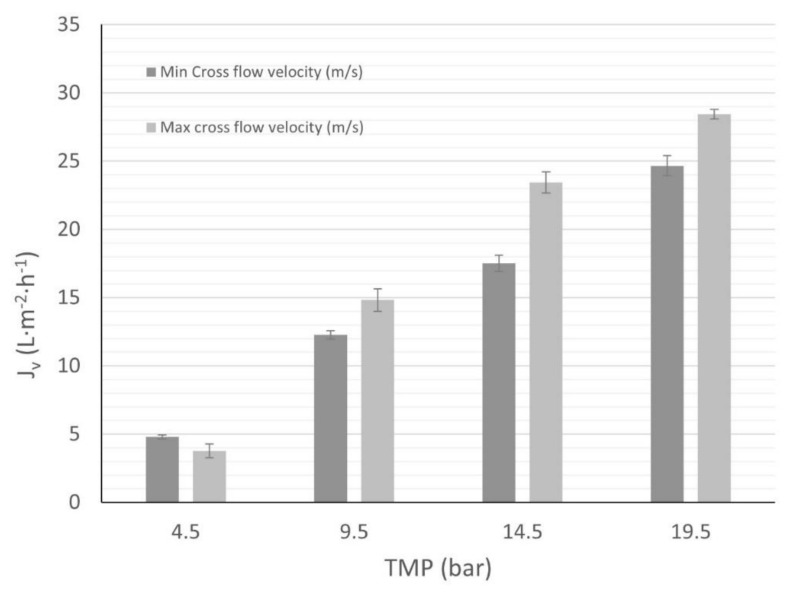
Wine lees (C1 solution) permeate flux behavior vs. cross-flow velocity. Minimum cross-flow velocity: 0.35 m·s^−1^. Maximum cross-flow velocity: *TMP* 4.5 bar, 0.42 m·s^−1^; *TMP* 9.5 bar, 0.54 m·s^−1^; *TMP* 14.5 bar, 0.66 m·s^−1^; and *TMP* 19.5 bar, 0.66 m·s^−1^.

Concerning the variation of $J_{v}$ with the cross-flow velocity, two velocities have been tested using solution C1 for each pressure. First, the same minimum velocity (0.35 m·s^−1^) has been used for all the pressures, resulting in an increase of the permeate flux with the pressure, as expected. Nevertheless, different maximum velocities were used due to the fact that our experimental setup did not allow for the same maximum cross-flow velocity for all operational conditions. The flux behavior was compared to the two velocities for each pressure and 298.15 K (Figure 8). The effect of velocity on the permeate flux was not remarkable at low pressure, thus indicating that concentration polarization CP was not serious. Although, when the pressure increased, the difference between the flux with minimum and maximum velocity raised as well. Higher values of cross-flow velocity contributed to removing the solutes from the membrane surface and reducing the concentration polarization effect. Hence, the concentration polarization CP could be a significant effect to include in the nanofiltration process.

**Figure 9:**
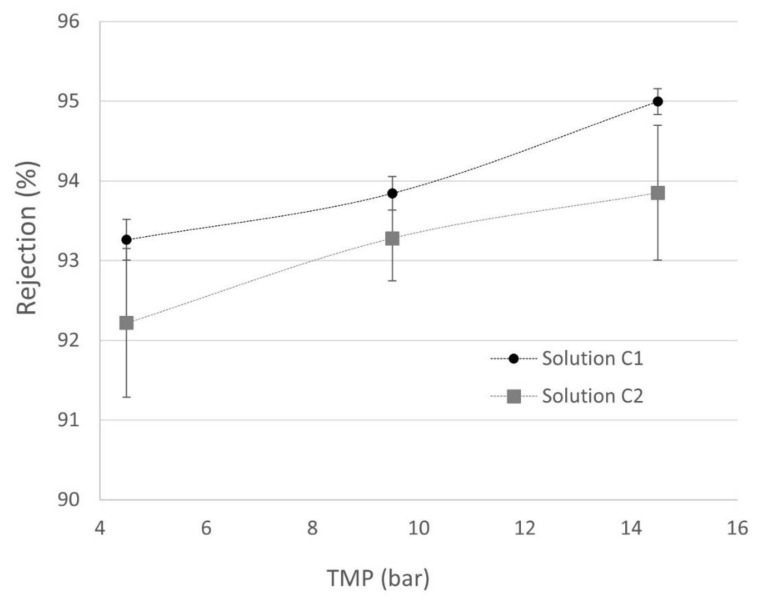
Observed solute rejection variation with feed polyphenol concentration (C1 and C2) vs. transmembrane pressure. ● rejection of solution C1 and ■ rejection of solution C2.

In regard to the observed polyphenols’ reject fraction (*R*) trend, the obtained results (Figure 9) show a weak dependence on the pressure, although this rejection slightly improved when transmembrane pressure increased according to the SKM model. The higher pressure is not plotted in Figure 9 because for C2 solutions, this operational parameter was discarded. Figure 4 shows that the improvement of polyphenol rejection at 19.5 bar related to 14.5 bar was not significant and energetically is less efficient.

However, there was a higher rejection when solution C1 was tested. In addition, the mathematical model also predicted that an increase in concentration leads to an enhanced CP phenomenon, which is observable in the displayed data. The trend of permeate concentration increasing with feed concentration was predictable, whereas in solution C1, permeate concentrations were observable around 100 mg tyrosol eq·L^−1^, and in solution C2, these permeate concentrations decreased until around 50 mg tyrosol eq·L^−1^. Real rejection differences between both concentrations could be produced by a fouling effect on the membrane, where polyphenols’ molecules are joined or agglomerated, forming a new layer with higher selectivity with respect to these species. 

### 3.4. Estimation of the Mass Transfer Coefficient Based on SKM

#### 3.4.1. Parameter Estimation

In this section, mathematical models 1 and 2 are fit to experimental permeate flux data, namely $J_{v\;exp}$. These models are based on Equation (3), which is indicated in the Introduction section. Model 1 describes the process when only water was tested and its predictions have to agree with the hydraulic permeability calculations of the Membrane Characterization: Pure Water Permeability section. Its dependence with the Arrhenius equation, model 2, was used for experiments when the problem dissolution was assayed. The non-linear regression problem was solved using Matlab R2020b and the Optimization Toolbox’s function lsqnonlin. Confidence intervals with a 95% significance level have been calculated with Statistics and Machine Learning Toolbox’s function nlparci. Additionally, for implicit model 2, Matlab’s function *fzero* has been used for obtaining $J_{v}$, assuming $J_{vinitial}=20\;L/\left(m^{2}\cdot h\right)$ as the starting guess value.

(4) $$\mathrm{Model}\;1:\;J_{v}=L_{p0}\cdot exp\left(-\frac{\Delta H}{R}\cdot \left(\frac{1}{T}-\frac{1}{T_{0}}\right)\right)\cdot \Delta P$$

(5) $$\mathrm{Model}\;2:\;J_{v}=L_{p0}\cdot exp\left(-\frac{\Delta H}{R}\cdot \left(\frac{1}{T}-\frac{1}{T_{0}}\right)\right)\cdot \left[\Delta P-\sigma \cdot R\cdot T\cdot \left(C_{f}-C_{p}\right)\cdot \;exp\left(\frac{J_{v}}{k}\right)\right]$$

A comparison between the experimental flux, $J_{v\;exp}$, and the theoretical or calculated one, $J_{v\;theor}$, using the fit parameters is plotted along Section 3.4 Estimation of the Mass Transfer Coefficient Based on SKM. The identity line, with a slope of 1, is also plotted as a reference for comparing both fluxes expected to be equal at the same operating conditions. The goodness of fit was measured using Equation (6) based on the Normalized Root Mean Square Error (NRMSE), which indicates the best goodness of fit with a maximum value of 100%. From a qualitative point of view, the goodness of fit is confirmed by the fact that the plotted points are around and near the identity line.

(6) $$Fit\left(\%\right)=100\cdot \left(1-NRMSE\right)=100\cdot \left(1-\frac{\sqrt{\sum_{i}{\left(J_{vexp}\left(i\right)-J_{vtheor}\left(i\right)\right)}^{2}}}{\sqrt{\sum_{i}{\left(J_{vexp}\left(i\right)-\bar{J_{vexp}\left(i\right)}\right)}^{2}}}\right)$$

where $\bar{J_{v\;exp}\left(i\right)}$ is the average of all experimental flux data.

#### 3.4.2. Parameter Estimation with Model 1

**Figure 10:**
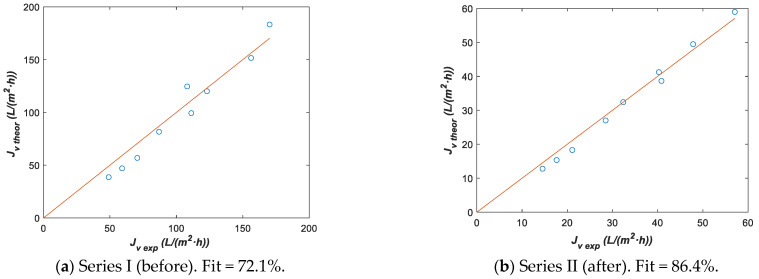
Theoretical vs. experimental data using fitting parameters from Table 8 with model 1. (**a**) Series I before lees waste and (**b**) Series II after lees waste and cleaning.

Model 1 has been fit to both experimental series I and II (Table 5) considering $T_{0}=293.15\;K$ with $L_{p0}$ and $\frac{\Delta H}{R}$ as fitting parameters. Initial guess values used in each optimization were $L_{p0\;initial}=1\;L/\left(m^{2}\cdot h\cdot \mathrm{bar}\right)\;$ and ${\frac{\Delta H}{R}}_{initial}=1\;K$, with the lower bounds $L_{p0\;lower}=0\;L/\left(m^{2}\cdot h\cdot \mathrm{bar}\right)\;$ and ${\frac{\Delta H}{R}}_{lower}=0\;K$, and upper bounds $L_{p0\;upper}=15\;L/\left(m^{2}\cdot h\cdot \mathrm{bar}\right)\;$ and ${\frac{\Delta H}{R}}_{upper}=\infty$. Fitting parameters with the corresponding 95% confidence intervals are shown in Table 8, while the comparison between $J_{v\;exp}$ and $J_{v\;theor}$ is plotted in Figure 10. For $L_{p0}$, the confidence interval was narrower than that corresponding to $\frac{\Delta H}{R}$, indicating the greater sensitivity of the former parameter in model 1. Thus, we can conclude that $\frac{\Delta H}{R}$ barely changes from series I to series II experiments. Nevertheless, a clear decrease in the $L_{p0}$ value was detected in series II and there was an agreement with the *L_p_* value for 293.15 K, as presented in Figure 5a,b from the above section.

#### 3.4.3. Parameter Estimation with Model 2

**Figure 11:**
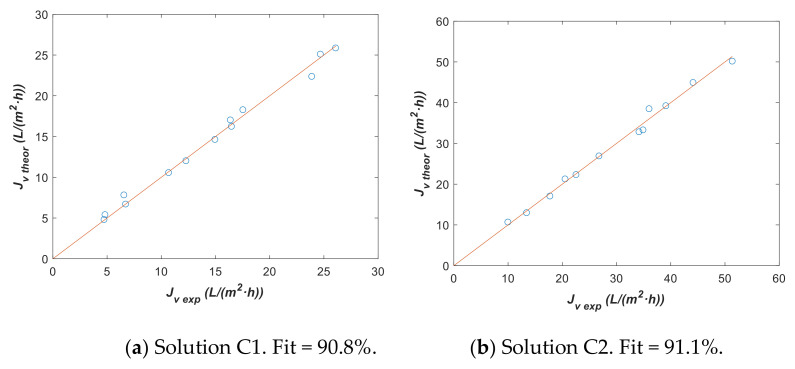
Theoretical vs. experimental data using fitting parameters from Table 10 with model 2. (**a**) Solution C1 and (**b**) solution C2.

The NF270 membrane was tested with problem solutions C1 and C2, and model 2 has been used for parameter estimation. Experimental data are shown in Table 9 at different operation conditions. Solution C1 has a greater concentration than solution C2 and fluxes with C1 are lower than those with C2. Assuming that σ=0.999 and considering, as a hypothesis, that mass transfer parameter k is not affected by temperature, fitting parameters $L_{p0}$, $\frac{\Delta H}{R}$ and k have been estimated using all experimental data from Table 9. The initial guess values, lower bounds, and upper bounds used were the same as those described for model 1, together with $k_{initial}=1\;L/\left(m^{2}\cdot h\right)$, $k_{lower}=0.1\;L/\left(m^{2}\cdot h\right)$, and $k_{upper}=\infty \;$. Table 10 contains fitting parameters with the corresponding 95% confidence intervals and Figure 11 shows a comparison between $J_{v\;exp}$ and $J_{v\;theor}$.

Figure 11 confirms a good agreement between the experimental flux data and theoretical values estimated with fitting parameters from Table 10. The confidence intervals for $L_{p0}$ and also for k were narrower than that of $\frac{\Delta H}{R}$, although the interval for k with solution C1 was the same order of magnitude of the fit parameter and it did not provide a conclusive value. This fact means that k with solution C1 has not been estimated with enough accuracy for comparing with k, corresponding to solution C2. Related to $L_{p0}$, a higher value of around twice was assigned to solution C2 and also a higher value for $\frac{\Delta H}{R}$ has been obtained, although the low sensitivity of this latter parameter pointed to not extracting too much information of this fact. However, there was a difference between calculated *L_p_*_0_ from solution C1 and C2 experimental data. This difference might indicate a change in the fouling on the membrane. A membrane cleaning with the pass of diluted solution C2 seems to cause this parameter to increase with respect to the permeability calculated in solution C1. This could indicate that when this proposed mathematical model is used, the effect over *L_p_*_0_ has to be considered.

**Figure 12:**
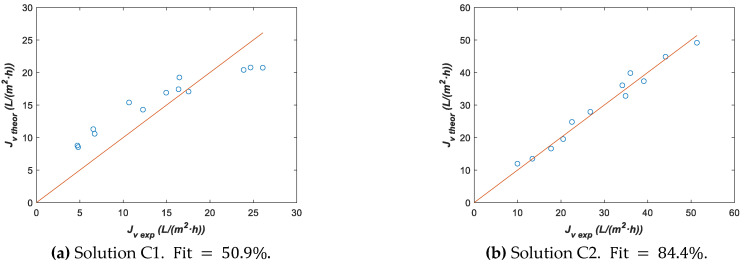
Theoretical vs. experimental data using fitting parameter from Table 11 with model 2 assuming $L_{p0}=2.8\;L/\left(m^{2}\cdot h\cdot \mathrm{bar}\right)$ and $\frac{\Delta H}{R}=3181\;K$. (**a**) Solution C1 and (**b**) solution C2.

It is interesting to note that $L_{p0}$ for solution C2 obtained with model 2 was similar to $L_{p0}$ for series II estimated with model 1, although for solution C1 it was lower. One could think that the fitting parameters estimated from series II with model 1 could be used in model 2 assuming that $L_{p0}$ and $\frac{\Delta H}{R}$ characterize the used membrane structurally and this information is independent of treating water or a concentrated solution. In this case, the only fitting parameter of model 2 that should be estimated would be k. Thus, let us assume the $L_{p0}=2.8\;L/\left(m^{2}\cdot h\cdot \mathrm{bar}\right)\;$ and $\frac{\Delta H}{R}=3181\;K$ parameters obtained with model 1 and series II for estimating only k with solutions C1 and C2, as well as model 2. Table 11 shows fitting parameter k with its 95% confidence interval and Figure 12 plots $J_{v\;theor}$ vs. $J_{v\;exp}$.

**Figure 13:**
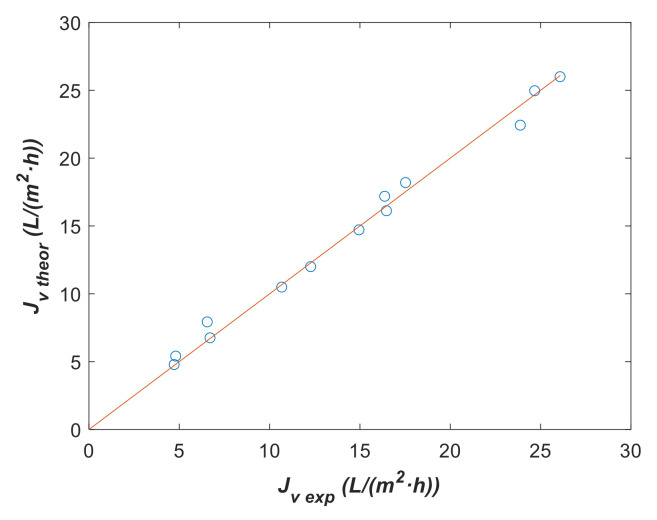
Theoretical vs. experimental data using fitting parameters from Table 12 with model 2 assuming $\frac{\Delta H}{R}=3181\;K$. Fit = 90.7%.

By contrasting k values from Table 10 and Table 11, we can affirm that these values are very similar for solution C2 and the goodness of fit (Figure 12b) still remains, although the $\frac{\Delta H}{R}$ values are different. This fact strengthens the idea about the low sensitivity of the parameter $\frac{\Delta H}{R}$ in models 1 and 2, thus we assumed $\frac{\Delta H}{R}=3181\;K$ in the following optimizations for reducing the number of fitting parameters and narrowing the corresponding confidence intervals. On the other hand, for solution C1, Figure 12a shows a poor fit due to the $L_{p0}$ value assumed in this optimization process, which means that for solution C1, it is not possible to consider the same $L_{p0}$ value estimated from series II with model 1, although, the *k* values in this fit were more consistent for experimental data. Thus, we have a fit again of model 2 to solution C1 experimental data but taking into account both $L_{p0}$ and k as fitting parameters (Table 12 and Figure 13). 

The results for the fitting parameters of solution C1 in Table 10 and Table 12 are similar and the goodness of fit is good enough, as shown in Figure 13. A slight reduction in the confidence interval for k was detected after diminishing the number of fitting parameters. All these fittings let us consider that there is not a clear effect on the k value of the type of solution tested with the membrane. However, when $J_{v}$ vs. *k* is calculated using the SKM model from a determined *k* value, the permeate flux remains almost constant, although *k* is increased. Thus, it is possible that the confidence intervals were very inaccurate due to this calculated plateau.

**Figure 14:**
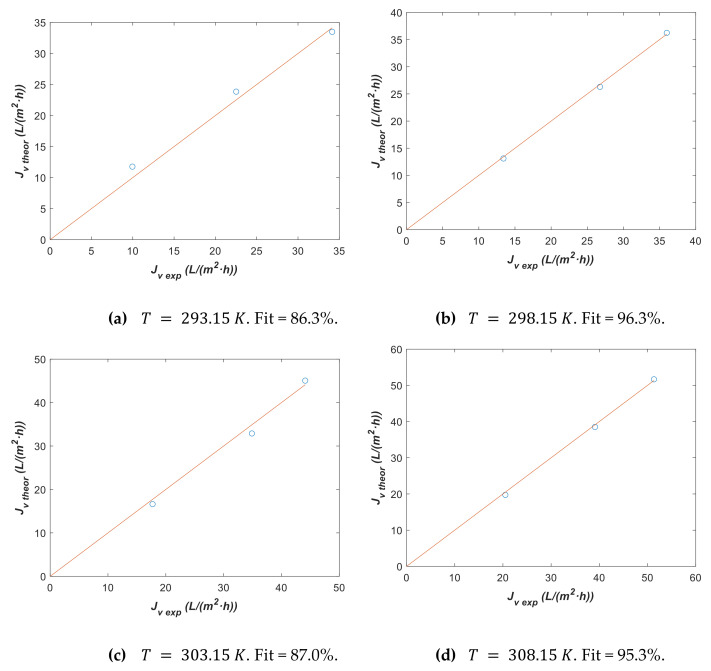
Theoretical vs. experimental data using fitting parameter from Table 13 with model 2 assuming $L_{p0}=2.8\;L/\left(m^{2}\cdot h\cdot \mathrm{bar}\right)\;$ and $\frac{\Delta H}{R}=3181\;K$ for solution C2 at different temperatures. (**a**) *T* = 293.15 K, (**b**) *T* = 298.15 K, (**c**) *T* = 303.15 K, and (**d**) *T* = 308.15 K.

Finally, we have assumed previously that k is not affected by temperature. In this case, we fit only k considering $L_{p0}=2.8\;L/\left(m^{2}\cdot h\cdot \mathrm{bar}\right)\;$ and $\frac{\Delta H}{R}=3181\;K$ with model 2 to solution C2 experimental data classified by temperature for contrasting this hypothesis. The fitting parameter, together with its confidence interval, is registered in Table 13 and $J_{v\;theor}$ vs. $J_{v\;exp}$ is plotted in Figure 14 for each temperature.

Figure 14 shows a goodness of fit adequate for all the temperatures. Additionally, Table 13 indicates a slight increase of k with temperature. Nevertheless, from a practical point of view, it can be better to use the same k value for all temperatures as has been demonstrated in Figure 12b where $k=13.3\;\pm \;1.2\;L/\left(m^{2}\cdot h\right)$ has been estimated. This trend for *k* agrees with experimental values.

## 4. Conclusions

The results presented in this work demonstrate the suitability of nanofiltration technology to recover valuable compounds such as polyphenols from wine lees. This process has two effects: first, a decrease in waste from the wine industry, and, on the other hand, a revalorization of them. When tyrosol solution, such as a reference compound of wine lees wastes, is treated, the NF90 membrane seems to be the best; however, when real wine lees wastes are treated, only NF270 shows enough capability to be used in the industry. The NF270 membrane offers acceptable permeate flux values and rejections at working pressures. Using real wine lees solutions for the industrial plant design is preferable to using a synthetic waste solution.

Polyphenol rejection was higher than 92% with permeate fluxes between 40.3 ± 0.8 and 57.1 ± 1.5. with a *TMP* of 19.5 bar. Furthermore, changes in *L_p_* were observed during the experimental setups, which appear to be mainly due to the fouling process and a change in the dissolution of products across the membrane. With regard to the hydrophilicity of the membrane, the wetting angle of the used membrane after water cleaning showed an increase from 19.3 to 43.5 and a fouling index of around 70%.

In addition, the Concentration Polarization (CP) appears when the polyphenol concentrations were taken into account. At low concentrations, the differences between water-pure and wine lees solution permeation fluxes maintain as constant around 20% in all temperatures. Nevertheless, when high concentrations of wine lees wastes are analyzed, the permeate fluxes’ differences between pure water and solution increase to 60%. The effect of velocity on the permeate flux is not remarkable at low pressure, thus indicating that concentration polarization was not serious; although, when the pressure increases, this CP has a significant effect to be included in the nanofiltration process. However, there is an increase in the real rejections when high concentrations of wine lees wastes are treated. This phenomenon is possibly due to polyphenols’ molecules being joined or agglomerated, forming a new layer with higher selectivity with respect to these species.

The SKM model parameters such as *k, L_p_*_0_, and *∆H/R* were fitted to the experimental data by non-linear regression. The goodness of fit of the SKM model can be considered adequate for an excellent description of the experimental data. This research shows that the *L_p0_* parameter can change during the nanofiltration treatment of wine lees (from 8.6 to 2.8 $L/m^{2}\cdot h\cdot \mathrm{bar}$), thus it has to be taken into account when the permeate flux is predicted. Moreover, the *k* parameter was not sometimes estimated with enough accuracy. Even so, it can be considered that the mathematical model adequately describes the nanofiltration membrane performance in the polyphenols’ recovery.

## Figures and Tables

**Table 1:** Literature search.

Research Engine	Accessed Date	Search	References
Web of Science	27 January 2022	polyphenol AND recovery AND wine	291
Web of Science	27 January 2022	polyphenol AND recovery AND wine AND membrane	21
Web of Science	27 January 2022	polyphenol AND recovery AND wine AND membrane AND lees	8

**Table 2:** Characteristics of the C1 wine lees sample.

Property	Value
pH	4.2 ± 0.2 ^a^
Conductivity (mS·cm^−1^)	3.1 ± 0.1
Tyrosol	Detected by HPLC [25]
Total polyphenols (mg tyrosol eq·L^−1^)	2036 ± 45

^a^ Standard deviation.

**Table 3:** Specifications of commercial nanofiltration and reverse-osmosis membranes.

Membrane	NF90	NF245	NF270	ESPA1
Membrane type	Nanofiltration	Nanofiltration	Nanofiltration	Reverse-osmosis
Membrane material	Thin-film composite polyamide	Thin-film composite polypropylene	Thin-film composite polypiperazine	Thin-film composite polyamide
Minimum salt rejection (%)	>97 ^a^	N/A ^b^	>97 ^a^	>98 ^c^
Permeate flow rate (m^3^·d^−1^)	2.6	N/A	3.2	2.84
MWCO (Da)	18—[26,27]	<300 [28]	20—[27,28]	N/A
Cl^−^ tolerance (ppm)	<0.1	ND ^d^	<0.1	<0.1
Maximum operating pressure (bar)	41	54.8	41	21
Maximum operating temperature (°C)	45	50	45	45

^a^ Data reported by the manufacturer based on the following conditions: 2000 ppm MgSO_4_ solution at 25 °C, 4.8 bar, and recovery of 15%. ^b^ Not available. ^c^ Data reported by the manufacturer based on the following conditions: 1500 ppm NaCl solution at 25 °C, 10.3 bar, and recovery of 10%. ^d^ Non-detectable.

**Table 4:** Permeate flux and rejection values for the experiments with tyrosol for the NF90 membrane.

Temperature (K)	*TMP* (Bar)	*J_v_* (L·h^−1^·m^−2^)	Rejection (%)
293.15	4.5	9.8 ± 1.0 ^a^	73.1 ± 6.2 ^a^
	9.5	14.4 ± 0.8	82.5 ± 0.9
	14.5	28.1 ± 0.7	55.7 ± 1.2
298.15	4.5	11.3 ± 0.9	70.3 ± 2.6
	9.5	18.1 ± 2.9	27.6 ± 1.9
	14.5	31.2 ± 0.2	55.7 ± 1.4
303.15	4.5	13.1 ± 1.3	59.3 ± 2.1
	9.5	18.6 ± 2.1	69.9 ± 2.6
	14.5	32.6 ± 4.3	62.9 ± 1.8

^a^ Standard deviation.

**Table 5:** Permeate flux values for the NF270 membrane to osmotic water before (series I) and after (series II) being exposed to wine lees wastes.

Temperature (K)	*TMP* (Bar)	*J_v_* (L h^−1^·m^−2^; Series I)	*J_v_* (L·h^−1^·m^−^^2^; Series II)
293.15	4.5	49.1 ± 1.0 ^a^	14.5 ± 0.9 ^a^
	9.5	87.0 ± 0.8	28.5 ± 0.9
	14.5	108.1 ± 0.7	40.3 ± 0.8
298.15	4.5	59.2 ± 0.9	17.7 ± 0.9
	9.5	111.2 ± 3.9	32.4 ± 0.9
	14.5	156.3 ± 0.2	47.9 ± 1.0
303.15	4.5	70.6 ± 1.3	21.1 ± 0.8
	9.5	123.0 ± 2.1	40.8 ± 1.1
	14.5	170.2 ± 4.3	57.1 ± 1.5

^a^ Standard deviation.

**Table 6:** Determination of the wetting angle for the NF270 membrane.

Membrane Condition	Wetting Angle (°)
New membrane	19.3 ± 0.1 ^a^
Cleaning with water	43.5 ± 0.7
Chemical cleaning	41.8 ± 0.6

^a^ Standard deviation.

**Table 7:** Determination of the fouling index (FI) for the NF270 membrane after experiments with wine lees residues.

*TMP* (Bar)	FI (%) 293.15 K	FI (%) 298.15 K	FI (%) 303.15 K
4.5	70.4	70.2	70.1
9.5	67.3	70.9	66.8
14.5	62.8	69.4	66.5

**Table 8:** Fitting parameters of model 1.

Fitting Parameter	Series I (before)	Series II (after)
$L_{p0}\;\left(L/\left(m^{2}\cdot h\cdot \mathrm{bar}\right)\right)$	8.6 ± 1.4	2.8 ± 0.2
$\frac{\Delta H}{R}\left(K\right)$	3428 ± 2011	3181 ± 1029

**Table 9:** Experimental data of membrane permeate flux, feed, and permeate concentrations for C1 and C2.

		Solution C1			Solution C2		
$P\;\left(bar\right)$	$T\;\left(K\right)$	$C_{f}\;$ $\left(mol/L\right)$	$C_{p}\;$ $\left(mol/L\right)$	$J_{v\;exp}\;$ $\left(L/\left(m^{2}\cdot h\right)\right)$	$C_{f}\;$ $\left(mol/L\right)$	$C_{p}\;$ $\left(mol/L\right)$	$J_{v\;exp}\;$ $\left(L/\left(m^{2}\cdot h\right)\right)$
4.5	293.15	1.47 × 10^−2^	7.03 × 10^−4^	4.7 ± 0.2 ^a^	5.45 × 10^−3^	4.74 × 10^−4^	10.0 ± 0.3 ^a^
4.5	298.15	2.18 × 10^−2^	14.65 × 10^−4^	4.8 ± 0.1	8.66 × 10^−3^	6.12 × 10^−4^	13.4 ± 0.6
4.5	303.15	1.47 × 10^−2^	11.10 × 10^−4^	6.7 ± 0.3	5.25 × 10^−3^	4.53 × 10^−4^	17.7 ± 0.4
4.5	308.15	1.47 × 10^−2^	11.69 × 10^−4^	6.5 ± 0.3	4.39 × 10^−3^	2.96 × 10^−4^	20.5 ± 0.5
9.5	293.15	1.47 × 10^−2^	8.18 × 10^−4^	10.7 ± 0.4	5.45 × 10^−3^	4.41 × 10^−4^	22.5 ± 0.9
9.5	298.15	2.25 × 10^−2^	12.40 × 10^−4^	12.3 ± 0.8	7.03 × 10^−3^	4.42 × 10^−4^	26.8 ± 1.3
9.5	303.15	1.47 × 10^−2^	9.64 × 10^−4^	14.9 ± 0.6	5.18 × 10^−3^	2.94 × 10^−4^	34.9 ± 0.9
9.5	308.15	1.47 × 10^−2^	10.35 × 10^−4^	16.4 ± 0.6	4.49 × 10^−3^	3.06 × 10^−4^	39.1 ± 1.0
14.5	293.15	1.47 × 10^−2^	6.24 × 10^−4^	16.5 ± 0.7	5.25 × 10^−3^	2.27 × 10^−4^	34.1 ± 1.1
14.5	298.15	2.53 × 10^−2^	10.48 × 10^−4^	17.5 ± 1.3	6.19 × 10^−3^	4.22 × 10^−4^	36.0 ± 2.2
14.5	303.15	1.47 × 10^−2^	8.65 × 10^−4^	23.9 ± 0.6	5.18 × 10^−3^	3.90 × 10^−4^	44.1 ± 1.7
14.5	308.15	1.47 × 10^−2^	8.51 × 10^−4^	26.1 ± 0.8	4.49 × 10^−3^	2.66 × 10^−4^	51.3 ± 1.5
19.5	298.15	1.95 × 10^−2^	7.18 × 10^−4^	24.7 ± 0.7			

^a^ Standard deviation.

**Table 10:** Fitting parameters of Model 2.

Fitting Parameter	Solution C1	Solution C2
$L_{p0}\;\left(L/\left(m^{2}\cdot h\cdot \mathrm{bar}\right)\right)$	1.2 ± 0.1	2.5 ± 0.2
$\frac{\Delta H}{R}\left(K\right)$	3056 ± 634	4529 ± 853
$k\;\left(L/\left(m^{2}\cdot h\right)\right)$	19.2 ± 20.5	13.0 ± 1.3

**Table 11:** Fitting parameter of model 2 assuming $L_{p0}=2.8\;L/\left(m^{2}\cdot h\cdot \mathrm{bar}\right)$ and $\frac{\Delta H}{R}=3181\;K$.

Fitting Parameter	Solution C1	Solution C2
$k\;\left(L/\left(m^{2}\cdot h\right)\right)$	6.2 ± 1.1	13.3 ± 1.2

**Table 12:** Fitting parameters of model 2 assuming $\frac{\Delta H}{R}=3181\;K$.

Fitting Parameter	Solution C1
$L_{p0}\;\left(L/\left(m^{2}\cdot h\cdot \mathrm{bar}\right)\right)$	1.2 ± 0.1
$k\;\left(L/\left(m^{2}\cdot h\right)\right)$	17.9 ± 13.6

**Table 13:** Fitting parameters of model 2 for solution C2 at different temperatures assuming $L_{p0}=2.8\;L/\left(m^{2}\cdot h\cdot \mathrm{bar}\right)\;$ and $\frac{\Delta H}{R}=3181\;K$.

Fitting Parameter	T=293.15 K	T=298.15 K	T=303.15 K	T=308.15 K
$k\;\left(L/\left(m^{2}\cdot h\right)\right)$	10.8 ± 5.1	11.0 ± 0.9	13.5 ± 4.0	14.5 ± 1.4

## Data Availability

The data presented in this study are available upon request from the corresponding author.
